# Rapid and Efficient Filtration-Based Procedure for Separation and Safe Analysis of CBRN Mixed Samples

**DOI:** 10.1371/journal.pone.0088055

**Published:** 2014-02-05

**Authors:** Mostafa Bentahir, Frederic Laduron, Leonid Irenge, Jérôme Ambroise, Jean-Luc Gala

**Affiliations:** 1 Biothreats Unit, Defense Laboratories Department (DLD), Belgian Armed Forces, Brussels, Belgium; 2 Centre de Technologies Moléculaires Appliquées, Institut de Recherche Expérimentale et Clinique, Université catholique de Louvain, Brussels, Belgium; 3 Chemical Analysis Laboratory, Defense Laboratories Department (DLD), Belgian Armed Forces, Brussels, Belgium; Ghent University, Belgium

## Abstract

Separating CBRN mixed samples that contain both chemical and biological warfare agents (CB mixed sample) in liquid and solid matrices remains a very challenging issue. Parameters were set up to assess the performance of a simple filtration-based method first optimized on separate C- and B-agents, and then assessed on a model of CB mixed sample. In this model, MS2 bacteriophage, *Autographa californica* nuclear polyhedrosis baculovirus (AcNPV), *Bacillus atrophaeus* and *Bacillus subtilis* spores were used as biological agent simulants whereas ethyl methylphosphonic acid (EMPA) and pinacolyl methylphophonic acid (PMPA) were used as VX and soman (GD) nerve agent surrogates, respectively. Nanoseparation centrifugal devices with various pore size cut-off (30 kD up to 0.45 µm) and three RNA extraction methods (Invisorb, EZ1 and Nuclisens) were compared. RNA (MS2) and DNA (AcNPV) quantification was carried out by means of specific and sensitive quantitative real-time PCRs (qPCR). Liquid chromatography coupled to time-of-flight mass spectrometry (LC/TOFMS) methods was used for quantifying EMPA and PMPA. Culture methods and qPCR demonstrated that membranes with a 30 kD cut-off retain more than 99.99% of biological agents (MS2, AcNPV, *Bacillus Atrophaeus* and *Bacillus subtilis* spores) tested separately. A rapid and reliable separation of CB mixed sample models (MS2/PEG-400 and MS2/EMPA/PMPA) contained in simple liquid or complex matrices such as sand and soil was also successfully achieved on a 30 kD filter with more than 99.99% retention of MS2 on the filter membrane, and up to 99% of PEG-400, EMPA and PMPA recovery in the filtrate. The whole separation process turnaround-time (TAT) was less than 10 minutes. The filtration method appears to be rapid, versatile and extremely efficient. The separation method developed in this work constitutes therefore a useful model for further evaluating and comparing additional separation alternative procedures for a safe handling and preparation of CB mixed samples.

## Introduction

The accidental release or deliberate use of chemical, biological, radiological and/or nuclear agents (CBRN) as weapons in warfare and terrorism threatens indistinctly the health of civilian and military personnel and may cause societal disruption. In recent years, several cases of chemical and biological terrorism have been recorded worldwide [1]. These incidents included the release of sarin nerve gas (Japanese cult Aum Shinrikyo 1995), the use of ricin [2], HIV-contaminated blood [3], *Salmonella* species and *Shigella* food contaminants [4] and the release of a powdery form of *B. anthracis* spores through the US post in 2001 [5]. Whereas these incidents were perpetrated using a single category of C- or B-agent, there is a growing concern that samples containing mixtures of hazardous biological, chemical and radioactive substances (CBRN mixed samples) might be used in the future.

For samples possibly contaminated with radio-elements, field analysis is preferred because existing screening methods are fast and reliable. However, samples suspected to contain both chemical and biological agents (CB mixed samples) pose a serious challenge as they need appropriate handling and preparation to allow subsequent safe analysis of the B- and C-components of the sample. When such samples are shipped to specialized laboratories, they require a first risk-assessment to prevent subsequent harmful exposure of personnel and contamination of the laboratory working space. The major issue for chemists is to remove and/or inactivate hazardous B-agents potentially contaminating the sample before performing subsequent chemical analysis. While inactivation can notably be achieved by high irradiation, this method is known to hinder the detection of irradiation-sensitive chemical agents [6]. Alternatively, inactivation may be achieved using chemical compounds or through removal of all B-agents by filtration. Conversely, biologists are concerned about the best way to decrease, or eventually to suppress specific chemical hazards in subsamples undergoing biological analysis. These issues are further complicated when the CB mixed sample is contained in a complex matrix such as powder or soil. On the top of that, it should be worth noting that the concomitant presence of C- and B-agents within the same sample could affect DNA-based identification methods through inhibition of DNA recovery [7].

In 2004, the Centers for Disease Control and Prevention (CDC) assessed the methods for safe handling, processing and analysis of mixed-threat environmental samples used in several laboratories in the US. It was concluded that there was an important deficiency on these matters (CBIAC report 2004: Assessment of protocols involving unknown samples). Besides the CDC, many other military organizations and civilian laboratories have drawn similar conclusions and pointed out the crucial need for reliable methods for safe handling and preparation of CBRN mixed samples (2007 and 2011 reports of 1^st^ and 2^nd^ NATO mixed sample laboratory exercises).

However, the development of innovative new procedures for separating CBRN mixed samples depends also on the availability of adequate simulants structurally and biologically mimicking genuine chemical and biological hazardous agents, respectively. In the current model, the bacteriophage MS2 [8], the *Autographa californica* nuclear polyhedrosis baculovirus (AcNPV) [9], *B. atrophaeus* and *B. subtilis* spores were used as biological simulants [10]. Ethyl methylphosphnic acid (EMPA) and pinacolyl methylphophonic acid (PMPA) are the products of the spontaneous hydrolysis of the chemical warfare agents (CWA) VX and GD (soman), respectively. They were used as surrogates for the corresponding chemical warfare agents [11]–[13].

The adequate pore-size for the filtration-based method was first selected on separate C- and B-agents. Further assessment was carried out on a model of CB mixed sample (RNA virus [MS2]) and chemical compounds [PEG-400, EMPA and PMPA]) in complex matrices such as sand and soil. To assess accurately the ultrafiltration efficiency, this study developed performance indicators allowing a precise quantification of the biological and chemical simulants in the filtrate and filter-retained fractions.

## Materials and Methods

### Biological Simulants Production

A seed stock of bacteriophage MS2 and *E. coli* F+ MC-4100/pOX38 host strain were obtained from Dr. Majdalani (Bethesda, USA). The *E. coli* host was a transformed strain with the pOX38 plasmid encoding for proteins involved in pilus formation which is an important structure required for MS2 virus infection. To propagate MS2, *E. coli* host strain was grown in liquid Luria broth at 37°C with shaking to an optical density of 0.2 at 600 nm. MS2 viruses were then added at a multiplicity of infection (MOI) of approximately 5 and the culture was incubated under kanamycin selection for 2 to 4 h until complete lysis of the bacterial host strain. Cell debris were removed by centrifugation. The resulting supernatant was subsequently filtrated through a 0.45 µm filter and the viral stock stored at 4°C. The MS2 viral titer of the cell lysate was determined by culture method using the double agar overlay phage assay (DAL) [14]. Briefly, a tenfold serial dilution containing initially 100 µL of the MS2 virus and 100 µL of an exponential culture of the host strain were rapidly mixed with 2.5 mL of a molten Luria broth top agar (0.5% agar). The mixtures were poured onto 20 mL of solid Luria broth plates containing 1.5% agar. After an overnight incubation at 37°C, the resulting plaques were counted and the virus titer was calculated taking into account the volume of virus inoculation and the dilution factor.

The recombinant *Autographa californica* nuclearpolyhedrosis virus (AcNPV) expressing *Aequorea victoria* green-fluorescent gene (GenBank: L29345.1) was purchased from AB vector (San Diego, California, USA). *Spodoptera frigiperda* (SF9) host cell line was purchased from Life Technologies (Carlsbad, California, USA) and maintained in TC100 medium containing 10% fetal calf serum at 28°C. For AcNPV production, a seed fraction of the viral stock was diluted in 15 ml of TC100 medium supplemented with 10% fetal calf serum to an MOI of 0.2. This mixture was subsequently added to a semiconfluent culture of SF9 monolayer cells grown in a T75 flask. An uninfected control culture was set in parallel to follow SF9 monolayer growth. The virus propagation was followed by monitoring GFP fluorescence by microscopy. Five days post-transduction, produced virus in the conditioned media was harvested, centrifuged to remove cellular debris and the resulting AcNPV was stored at 4°C. *Bacillus subtilis* and *Bacillus atrophaeus* (ATCC 9372) spores were purchased from ArTechno (Liege, Belgium) and Liofilchem (Roseto degli Abruzzi, Italy), respectively.

### PCR Assay Design and Conditions

Two specific qPCR assays were developed to detect and quantify the number of MS2 and AcNPV genomes in various test samples. Primers were designed using Primer 3 algorithm [15]. For the amplification of MS2 bacteriophage, the extracted phage RNA was first retro-transcribed into cDNA using 1411-MS2-R primer (5′-AAGTTGCTTGGAGCGACAGT) and VersoTM cDNA synthesis kit (Thermo Scientific, Surrey, UK) according to the manufacturer’s instructions. A specific pair of primers 430-MS2-F: 5′-TGCTACAGCCTCTTCCCTGT and 681-MS2-R: 5′-ATCTTCGTTTAGGGCGAGGT was then used to amplify a 250 bp DNA fragment within the gene coding for MS2 bacteriophage assembly protein. Similarly, a pair of primers (452-Bac-F: 5′-ACATCATGGCAGACAAACCA and 696-Bac-R: 5′-GCCATGTGTAATCCCAGCAG) specific to the GFP encoding gene, generated a DNA fragment of 245 bp from GFP-recombinant AcNPV genomic DNA used as template for amplification.

The qPCR assay was carried out in 25 µL reaction volume containing 12.5 µL of 2x power SYBR Green master mix (Life technologies, Carlsbad, California, USA), 1.5 µL of each primer (300 nM) and 2 µL of AcNPV DNA or 2 µL of MS2 bacteriophage cDNA. The reaction was initiated at 50°C for 2 min, and 95°C for 10 min followed by 40 cycles of denaturation at 95°C for 15 s and annealing at 60°C for 1 min and extension for 30 seconds at 72°C. Each sample was tested in triplicate and data were recorded as Cycle threshold (Ct) on a TaqMan 7900HT Sequence Detection System (Life Technologies, Carlsbad, California, USA), using the analytical software from the same manufacturer.

To quantify the copy number of MS2 phage and AcNPV genomes by the qPCR assays, DNA fragments obtained after PCR amplification of MS2 and AcNPV nucleic acids were cloned into a pCR4 vector (Life Technologies, Carlsbad, California, USA) and transformed into a One-Shot TOP10 electro-competent *E. coli* according to the manufacturer’s instructions. The resulting recombinant plasmids (pCR4-MS2 and pCR4-AcNPV) were isolated from positive clones using the plasmid Midi Kit (Roche, Basel Switzerland) and inserts were verified by sequencing. The MS2 phage and AcNPV standard curves were created from 10-fold diluted recombinant plasmids pCR4-MS2 and pCR4-AcNPV assaying 2×10^5^ down to 2×10^1^ copies per PCR reaction as calculated from plasmid concentration (OD 260 nm). Each serial dilution was tested in triplicate and in three independent experiments. Plotting the cycle threshold values (Ct) against the log_10_ of the copy number generated a standard curve from each plasmid and allowed the accurate quantification of MS2 phage and AcNPV in various samples.

### Filtration

Various nanoseparation centrifugal devices bearing polyethersulfone membranes and displaying a pore size cut-off of 30, 100, 300 kD and 0.2 or 0.45 µm were purchased from Pall life science (Ann Arbor, MI, USA). The ability of various filters to retain bacteriophage MS2, AcNPV, *B. atrophaeus* or *B. subtilis* spores after filtration was assessed. Briefly, each biological simulant was prepared in 400 µL of Tris-Mg-Ca buffer (10 mM tris-HCl pH 7.4, 5 mM CaCl2 and 10 mM MgCl_2_), applied to various filter devices and centrifuged up to 5 min at 12000 g at room temperature. In these conditions, the totality of the test sample passed through the filtration membrane. Subsequently, culture-based assays were used to measure first the amount of each biological simulant in 100 µL of the filtrate and in an equal volume of an original non-filtrated sample containing approximately 10^6^, 10^7^ and 10^8^/ml of bacteriophage MS2, *B. atrophaeus* and *B. subtilis* spores. Next, q-PCR assays were used to assess filtration of bacteriophage MS2 and AcNPV through various filters in a second step. Following filtration of 400 µL containing approximately 10^8^ genome copies of MS2 or AcNPV, 200 µL of each test sample was used for nucleic acid extraction. The log_10_ reduction capacity of each filter for each biological simulant was calculated by subtracting the log_10_ number value measured in the filtrate from that measured in the original non-filtered sample.

### Recovery of MS2 Bacteriophage and AcNPV from Sand and Soil Matrices

Three various extraction kits were evaluated for the reproducible and efficient extraction of nucleic acid and the optimal recovery of MS2 bacteriophage and AcNPV from liquid, sand or soil matrices. The kits tested included Invisorb (Startec, Berlin, Germany), the NucliSens® miniMag semi-automated apparatus (Biomérieux Inc., Boxtel, The Netherlands) and the fully automated system EZ1 (Qiagen, The Netherlands) according to the manufacturer’s instructions. Briefly, 50 µL of the viral stock was diluted in 950 µL of Tris-Mg-Ca buffer or directly spiked in 0.25 g of sand or soil. Samples spiked with the viral simulant were allowed to dry for 1 h. Tris-Mg-Ca buffer (950 µL) was used to recover viruses from solid matrices by vortexing and insoluble material was removed by filtration through a 5 µm cut-off filters. Subsequently, 200 µL of each test sample was used for nucleic acid extraction. Elution was carried out in 60 µL of nuclease-free buffer. MS2 virus RNA was reverse transcribed into cDNA prior to PCR amplification. Data were compared for identifying the best extraction method. Virus recovery and nucleic acid extraction from soil matrix was optimized according to a method using glycine buffer (0.25 M, pH 9). For further optimization, this buffer was supplemented either with 1% BSA, 0.15, 0.5 M NaCl or a combination of BSA and salt.

The soil matrix used for spiking biological and chemical simulants originated from Namibia. This matrix had a dry mass of 97.4% at 105°C and contained 19.4, 61 and 19.6% of clay, silt and sand, respectively. The sand matrix contained 98.8, 0.71 and 0.49% of sand texture particles, silt and clay, respectively.

### Chemical Simulants

Polyethylene glycol 400 (PEG-400), ethyl methylphosphonic acid (EMPA) and pinacolyl methylphophonic acid (PMPA) were purchased from Sigma-Aldrich (St-Louis, USA). The water soluble EMPA and PMPA compounds arise from the spontaneous hydrolysis of chemical warfare agents (CWA) VX and GD (soman), respectively. For the quantification of PEG-400, EMPA and PMPA chemical compounds, detection methods based on liquid chromatography separation coupled to LC/TOFMS were developed. Briefly, serial dilutions of each chemical compound were prepared in water and injected into a C18 column (3×15 mm). The chromatographic separation of PEG-400 dilutions was performed at 25°C and at a flow rate of 0.2 mL/min using a gradient of 60 to 40% of solvent A (10 mM formic acid in water) while solvent B contained 10 mM formic acid prepared in acetonitrile. The ESI positive mode was selected and peak area was calculated based on the base peak chromatogram (BPC). The chromatographic separation of EMPA and PMPA compounds was performed at 40°C using a gradient 80–50–80% of solvent A. The ESI negative ion mode was selected. The peak area was calculated based on the EIC (extracted ion chromatogram) at 123.01±0.05 m/z for EMPA and 179.08±0.05 m/z for PMPA.

### CB Mixed Sample Models

To mimic CB mixed samples, bacteriophage MS2 simulant and chemical warfare agent surrogates were mixed and spiked either in a liquid, sand or soil matrix. In the first model (CB mixed sample n°1), 20 µL of MS2 viral stock containing 5×10^8^ genome copies were mixed together with 0.125, 0.25, 0.5 or 1% of PEG-400 in a 1 mL final volume of Tris-Mg-Ca buffer (liquid matrix). The second CB mixed sample model (CB mixed sample n°2) was prepared by mixing 20 µL of a 10-fold dilution of MS2 viral stock with 25 µg/mL of EMPA and PMPA. Mixed samples were diluted directly in 980 µL of glycine buffer (liquid matrix) or spiked either in a 0.25 g of sand or soil matrix. The recovery from sand or soil matrices was carried out by adding 980 µL of glycine buffer and subsequent prefiltration through a 5 µm cut-off filter. Separation was carried out by filtration through a 30 kD cut-off filter. The amount of biological and chemical simulants was then measured in the filtrate and the filter-retained fractions, respectively and compared with the total amounts of biological and chemical surrogates in a control sample analyzed before filtration.

### Statistical Analysis

Statistical analyses were performed using R software for statistical computing. Intra- and inter-assay coefficient of variations (CV) was calculated for qPCR and LC/TOF quantitative assays over the whole range of concentrations. For qPCR assays, CV were computed from corresponding copy numbers, as recommended in the MIQE Guidelines [16].

Percentages of recoveries were compared between types of matrix (*i.e.* liquid, sand and soil) and between extraction methods by using the non-parametric one-tailed Wilcoxon test.

Statistical tolerance intervals (95%/95%), which correspond to intervals containing the value of 95% of all units with a probability of 95%, were also used to characterize the filtrate and retained fractions of CB mixed samples.

## Results

### Quantification of Biological and Chemical Simulants

**Figure 1:**
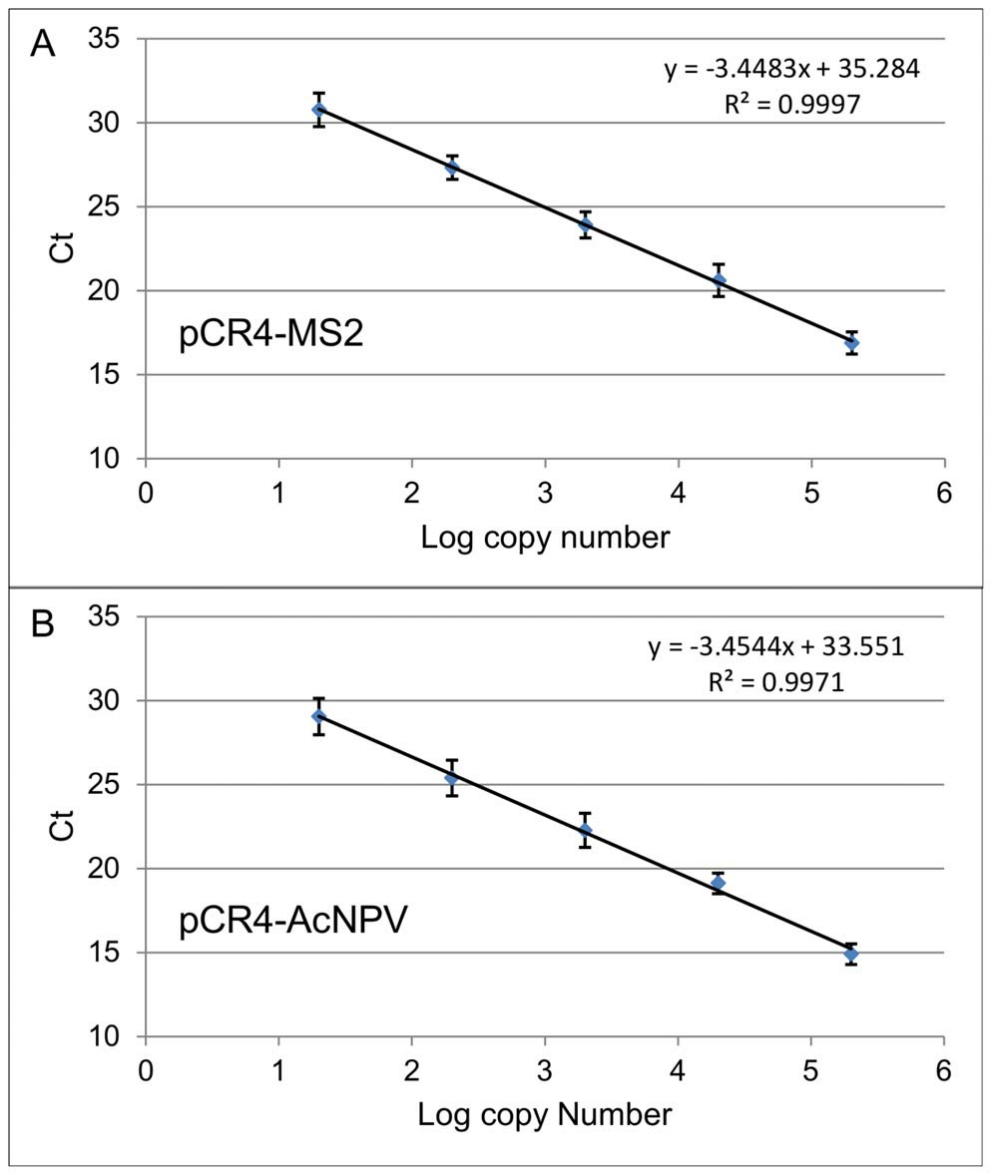
Standard curves. qPCR standard plasmid DNA dilution curves from: (A) recombinant MS2 bacteriophage (pCR4-MS2), (B) AcNPV (pCR4-AcNPV). Mean Ct values (Y axis) are plotted against logarithm (log_10_) of virus genome copies number. Each data point represents Ct average with standard deviation (SD, n = 3).

Approximately 100 mL of bacteriophage MS2 and recombinant AcNPV were produced at a titer of 10^11^ and 10^8^ viral particles/mL, respectively, as assessed by double layer agar culture method and qPCR. For detecting and quantifying MS2 and AcNPV, two specific qPCR assays using SYBR Green were developed. The specificity was confirmed on a panel of irrelevant DNA and RNA templates extracted from various unrelated viruses and bacteria (data not shown). The pCR4-MS2 and pCR4-AcNPV recombinant plasmids containing a MS2- and AcNPV-specific insert, respectively, were used to construct calibration curves. These were used to determine the absolute number of genome copies present in the samples (Fig. 1A and 1B). The qPCR assays displayed a dynamic range of at least five logs. The MS2 and AcNPV limit of detection was approximately 20 genome copies (∼46 fg), which corresponds to a Ct value of 30.8 and 29.1, respectively. The detection of 2 genome copies of each virus was stochastic at a Ct value higher than 33. Accordingly, samples with Ct values higher than 33 were considered to be negative. Intra- and inter-assay CV for pCR4-MS2 and pCR4-AcNVP qPCR results ranged from 15 to 26%, and 14 to 38%, respectively (Table 1).

**Table 1:** Quantification methods of biological (MS2 and AcNPV) viral agents and chemical (EMPA and PMPA) compounds: results and Coefficient of Variation (CV).

Coefficient of Variation CV (%)									
q-PCR	Copy number				2*10^5^	2*10^4^	2*10^3^	2*10^2^	2*10^1^
	pCR4-M2			Intra-assay	14.96	17.76	12.06	11.68	26.13
				Inter-assay	17.84	19.14	21.59	26.62	26.85
	pCR4-AcNPV			Intra-assay	13.76	14.73	19.73	17.98	35.95
				Inter-assay	20.80	18.92	30.59	21.47	38.37
LC/TOFMS	Concentration range (µg/ml)				0.1	0.25	0.5	1	
			Intra-assay		nd	nd	nd	nd	
			Inter-assay		5.24	9.60	7.83	4.38	
			Intra-assay		nd	nd	nd	nd	
			Inter-assay		2.45	2.57	3.32	2.64	
LC/TOFMS	Concentration range (%)				0.125	0.25	0.5	1	
		Intra-assay			5.09	6.81	6.96	12.69	
		Inter-assay			13.82	15.43	18.45	14.05	

nd = not determined (technical replicates not used in the experiment).

**Figure 2:**
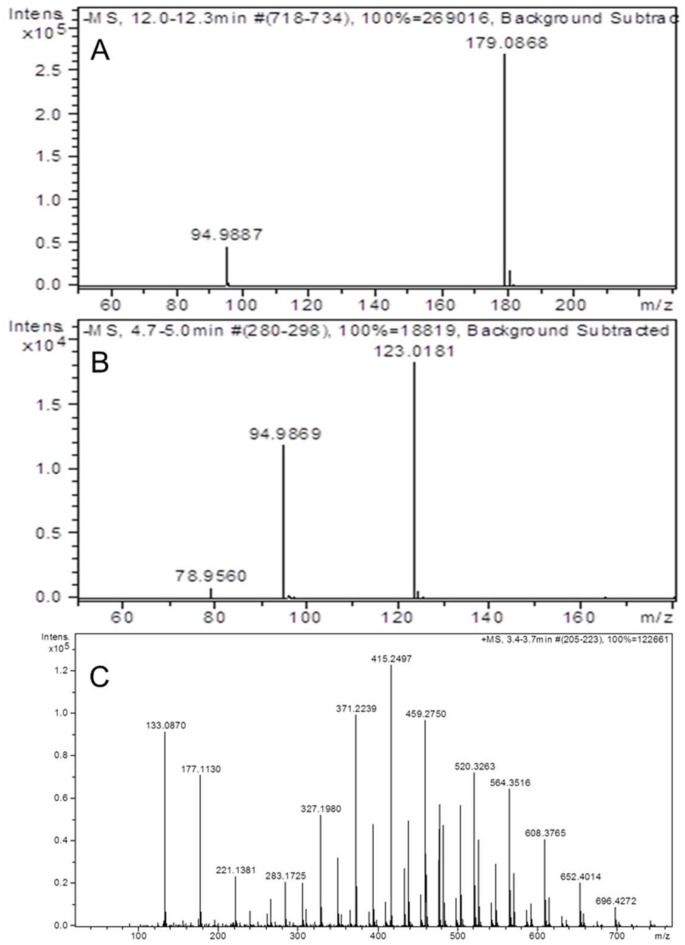
Mass spectra. Representative mass spectra for EMPA and PMPA surrogates and PEG-400 after liquid chromatography analysis coupled to LC/TOFMS. The ESI negative ion mode was used. Measurement of peak areas of PMPA and EMPA was based on the EIC at 179.08±0.05 m/z (A) and at 123.01±0.05 m/z (B), respectively. (C) Mass spectrum of PEG-400 was assessed with the ESI positive ion mode while measurement of the peak area was based on the BPC.

Detection and quantification of the chemical compounds PEG-400, EMPA and PMPA were carried out using LC/TOFMS (Fig. 2). The detection limit for EMPA and PMPA was 25 ng/mL and 10 ng/mL, respectively. For PEG-400 quantification, the intra- and inter-assay CV ranged from 5 to 13% and from 13 to 18%, respectively. In the range of EMPA and PMPA concentrations that were quantified, CV was lower than 5 and 10%, respectively (Table 1).

### Characterization of the Filtration Membranes

Culturing the resulting filtrates showed that all filtration devices achieved retention of more than 10^6^ and 10^7^ spores per reaction of *B. atrophaeus* and *B. subtilis spores*, respectively. A complete retention of MS2 virus was only achieved by using the 30 kD cut-off filter as demonstrated by the DAL method (Table 2) [14].

**Table 2:** *Bacillus atrophaeus*, *Bacillus subtilis* spores and MS2 virus filtration data as assessed by culture methods.

	Spores and MS2 titer before and after filtration					
	Before Filtration	After filtration				
		0.45 µm	0.22 µm	300 kD	100 kD	30 kD
*B. atrophaeus* (CFU)	2.53±0.12*(10^6^)	0	0	0	0	0
*B. subtilis* (CFU)	34.30±1.53*(10^6^)	0	0	0	0	0
*MS2* virus (PFU)	9.50±2.12*(10^4^)	nd	5.50±0.71*(10^4^)	8.15±0.21*(10^3^)	9.80±1.13*(10^2^)	0

nd = not determined (condition not tested), ± indicate SD (n = 3).

**Figure 3:**
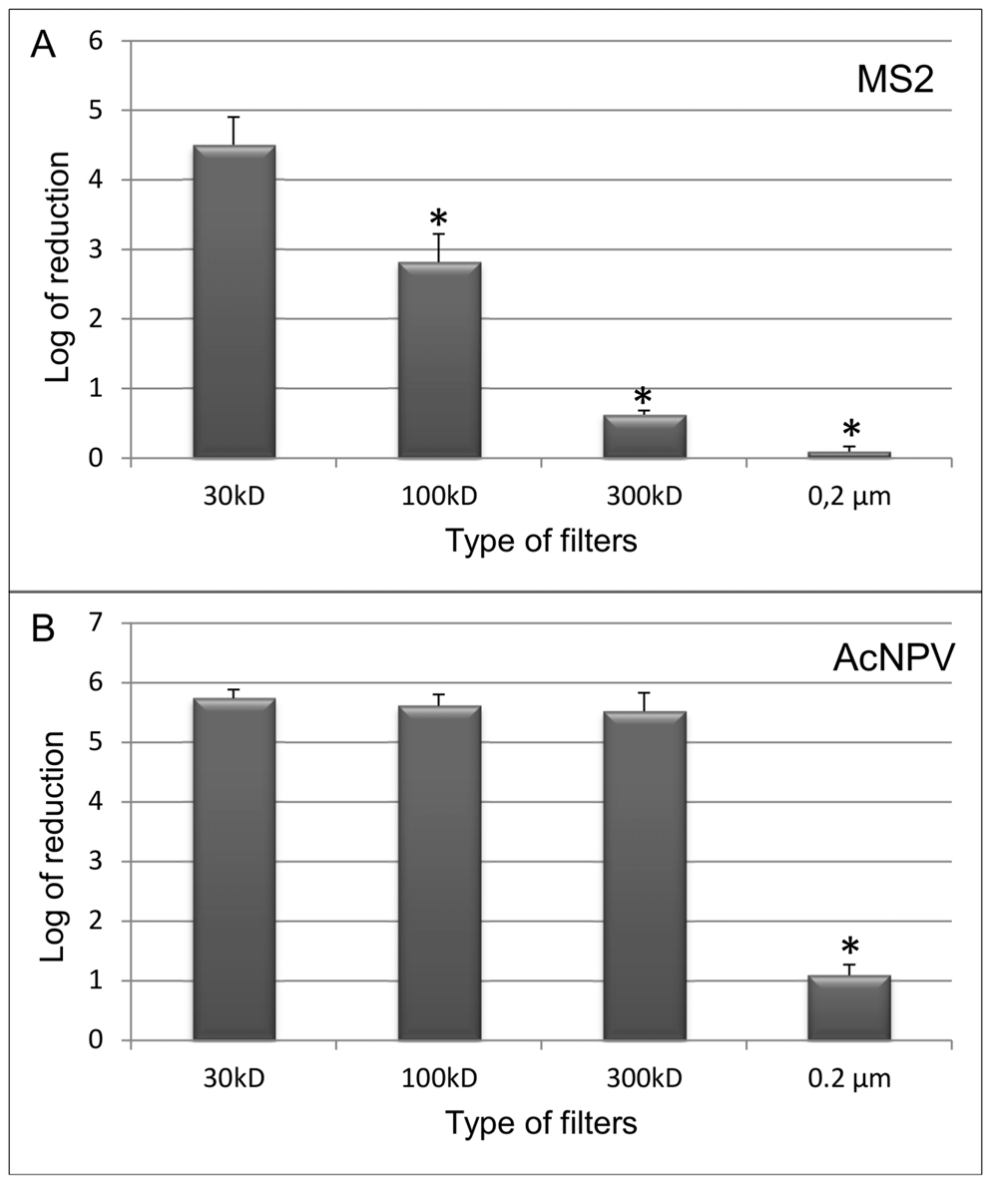
Bacteriophage MS2 and AcNPV filtration data. Log_10_ reduction of bacteriophage MS2 and AcNPV after filtration through various filter types. The virus titer was determined by qPCR on the filtrate and an unfiltered control sample. One-tailed Wilcoxon test was used to assess that log_10_ reduction in the filtrate of 100, 300 kD or 0.2 µm filters was significantly lower than that obtained with a 30 kD filter (*p = 0.05). (A) Mean log_10_ reduction of MS2 virus and SD (n = 3); (B) Mean log_10_ reduction and SD (n = 3) of AcNPV.

When assessing the filtration process by qPCR, the 30 kD cut-off filter appeared to be the most effective in retaining both MS2 and AcNPV viral agents (Fig. 3A and 3B). Indeed, qPCR data obtained on the total genomic material of MS2 bacteriophage showed the filter to reduce MS2 viral particles by a 4.50 (±0.39) log_10_ factor in the filtrate, which corresponds to a retention capacity higher than 99.99% (Fig. 3A). Similar results were also obtained when testing AcNPV. A log_10_ reduction capacity of 5.74 (±0.14), 5.61 (±0.19) and 5.52 (±0.30) was demonstrated for 30,100 and 300 kD cut-off filters, respectively (Fig. 3B).

### Recovery of MS2 Bacteriophage and AcNPV from Sand and Soil Matrices

**Figure 4:**
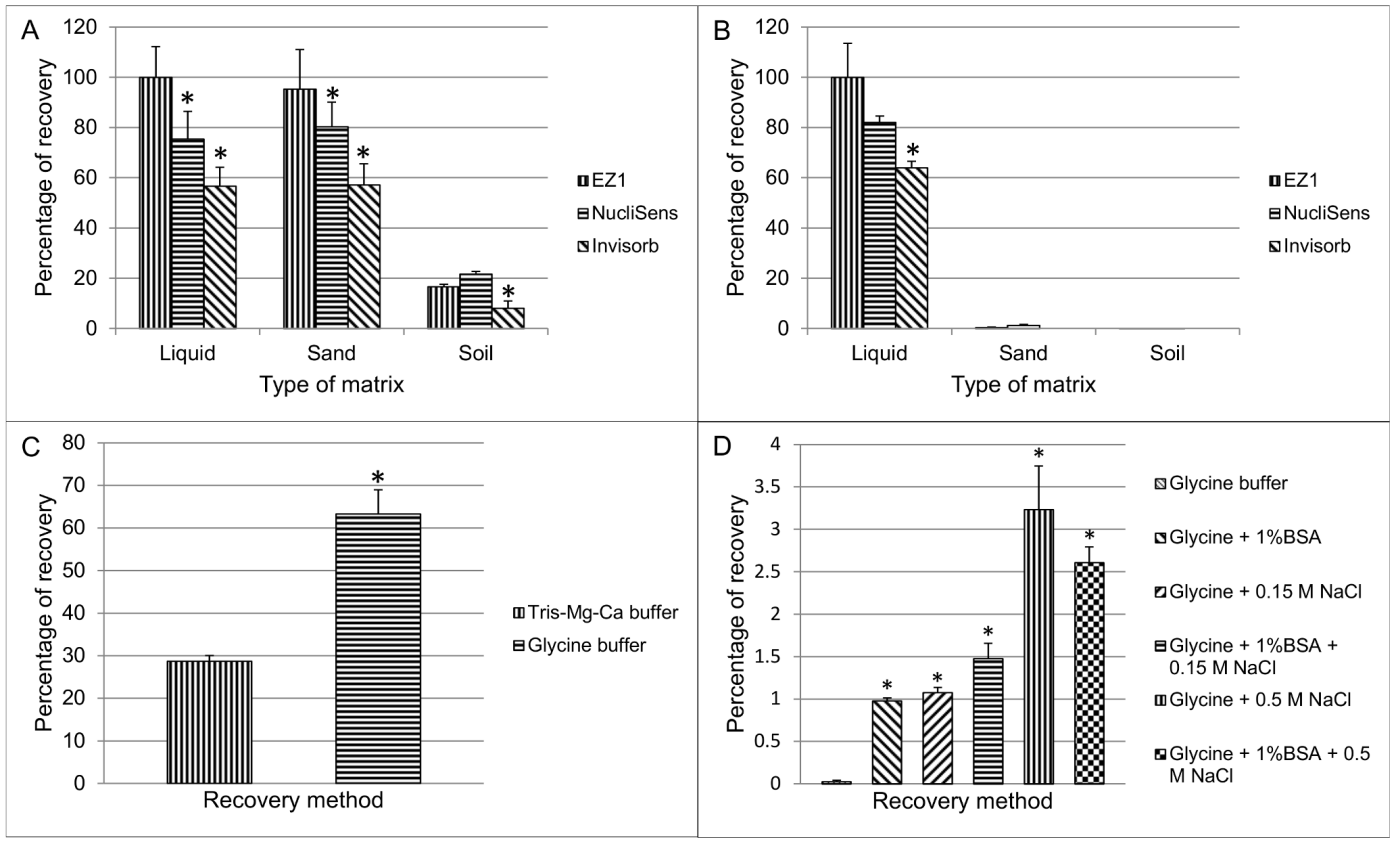
Comparative efficiency of MS2 and AcNPV viral simulants recovery. Recovery and extraction efficiencies were assessed by qPCR. Tris-Mg-Ca buffer (A and B) is used to recover viruses spiked in different matrices and to extract nucleic acids using different extraction kits, as indicated. One-tailed Wilcoxon test was used to assess if extraction efficiency was significantly lower with NucliSens and Invisorb as compared to EZ1 extraction kit (*p = 0.05). Mean percentage and SD (n = 3) of MS2 RNA (A) and AcNPV DNA extraction efficiency (B) from a liquid, sand or soil matrix after data normalization with the EZ1 extraction kit. (C and D) virus recovery and extraction from a soil matrix using NucliSens extraction kit, and assessing the impact of different types of recovery buffers, as indicated (one-tailed Wilcoxon, *p = 0.05). (C) Mean percentage and SD (n = 3) of MS2 RNA extraction comparing the effect of glycine and Tris-Mg-Ca recovery buffers. (D) Mean percentage and SD (n = 3) of AcNPV DNA extraction: for virus recovery, glycine buffer alone was compared to glycine buffer supplemented with BSA, NaCl or both.

Before carrying out the separation of CB mixed sample models after spiking experiments, MS2 and AcNPV recovery and extraction from simple liquid or complex matrices (sand and soil) were first assessed using three different extraction kits. The type of matrix and extraction kit influenced the recovery yield. In a liquid or sand matrix, extraction of MS2 RNA was optimal with EZ1 and, to a lesser extent, with NucliSens kit while extraction using the Invisorb kit appeared to be the least efficient method (Fig. 4A). However, the recovery and extraction of spiked MS2 bacteriophages into a soil matrix was much less satisfactory (Fig. 4A). For the latter, the recovery and extraction efficiency of bacteriophage MS2 was indeed 8%, 16% and 21% of the total amount of spiked virus with Invisorb, EZ1 and NucliSens extraction kit, respectively (Fig. 4A). Likewise, AcNPV recovery and extraction from a liquid matrix was also optimal using either EZ1 or NucliSens extraction kits whereas Invisob kit was the less efficient method (Fig. 4C). When spiked into a solid matrix (either sand or soil), AcNPV extraction failed nearly completely, irrespective of the extraction kit (Fig. 4C).

In order to optimize MS2 and AcNPV recovery from a soil matrix, a glycine buffer at pH 9 supplemented or not with either BSA, NaCl or both, was used for virus removal whereas the NucliSens kit was used for extraction. MS2 virus recovery and extraction was substantially improved when using glycine rather than tris-Mg-Ca buffer (Fig. 4B). Regarding AcNPV extraction, using glycine buffer followed by NucliSens extraction was the less efficient method. When the glycine buffer was supplemented either with 1% BSA or 0.15 M NaCl, the virus recovery was improved giving an extraction efficiency of ∼1% of the total amount of spiked virus (Fig. 4D). Increasing salt concentration up to 0.5 M improved significantly (up to 3%) AcNPV recovery (Fig. 4D) whereas adding both BSA (1%) and NaCl at either 0.15 or 0.5 M failed to further improve viral recovery rate (Fig. 4D). Considering speed and efficiency, the work was pursued further using glycine buffer and NucliSens kit for viral recovery and nucleic acid extraction, respectively.

### Separation of CB Mixed Sample Models

**Figure 5:**
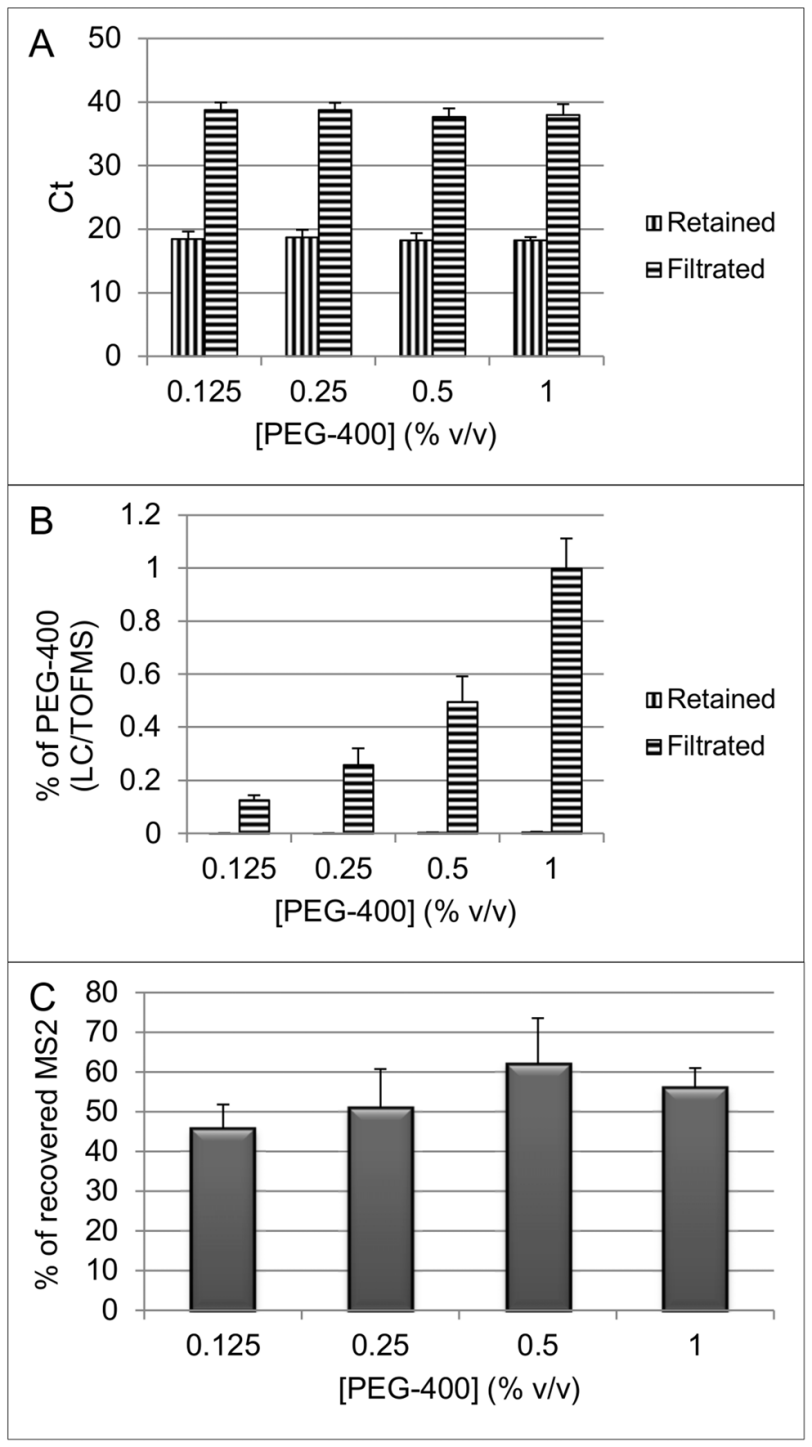
MS2 titers and PEG-400 concentrations after separation of CB mixed sample n°1. A fixed amount of MS2 bacteriophage (5×10^8^ genome copies in 20 µL) was mixed with increasing amounts of PEG-400. After filtration through a 30 kD filtration device, MS2 bacteriophage titers and PEG-400 concentration were measured by qPCR and LC-TOFMS, respectively. RNA extraction was carried out using NucliSens kit. (A) Mean Ct and SD (n = 3) values in filtrate and retained fractions. (B) Average percentages and SD (n = 3) measured in filtrate and retained fractions. (C) Mean percentage and SD (n = 3) of MS2 RNA recovery from filter and retained fractions.

The separation efficacy of C- and B-compounds from two CB reconstituted mixed samples was assessed using 30 kD ultrafiltration membranes. In CB mixed sample n°1, an increasing percentage of PEG-400 and a fixed amount of MS2 bacteriophages were mixed together. The filter-retained and filtrate fractions of C- and B-compounds were both analyzed using LC/TOFMS and qPCR, respectively. The MS2 fraction was retained almost completely on the filter membrane as demonstrated by mean Ct values of the filtrate higher than the intercept of the MS2 calibration curve corresponding to one genome copy (Fig. 5A). Log_10_ reduction of almost 5 (corresponding to more than 99.99% retention) was obtained for this mixed CB model sample irrespective of the PEG-400 concentration, whereas a proportional and almost complete (∼99%) distribution of PEG-400 compound was recovered from the filtrate (Fig. 5B). The filter retained MS2 fraction varied between 45 and 61% (Fig. 5C).

**Figure 6:**
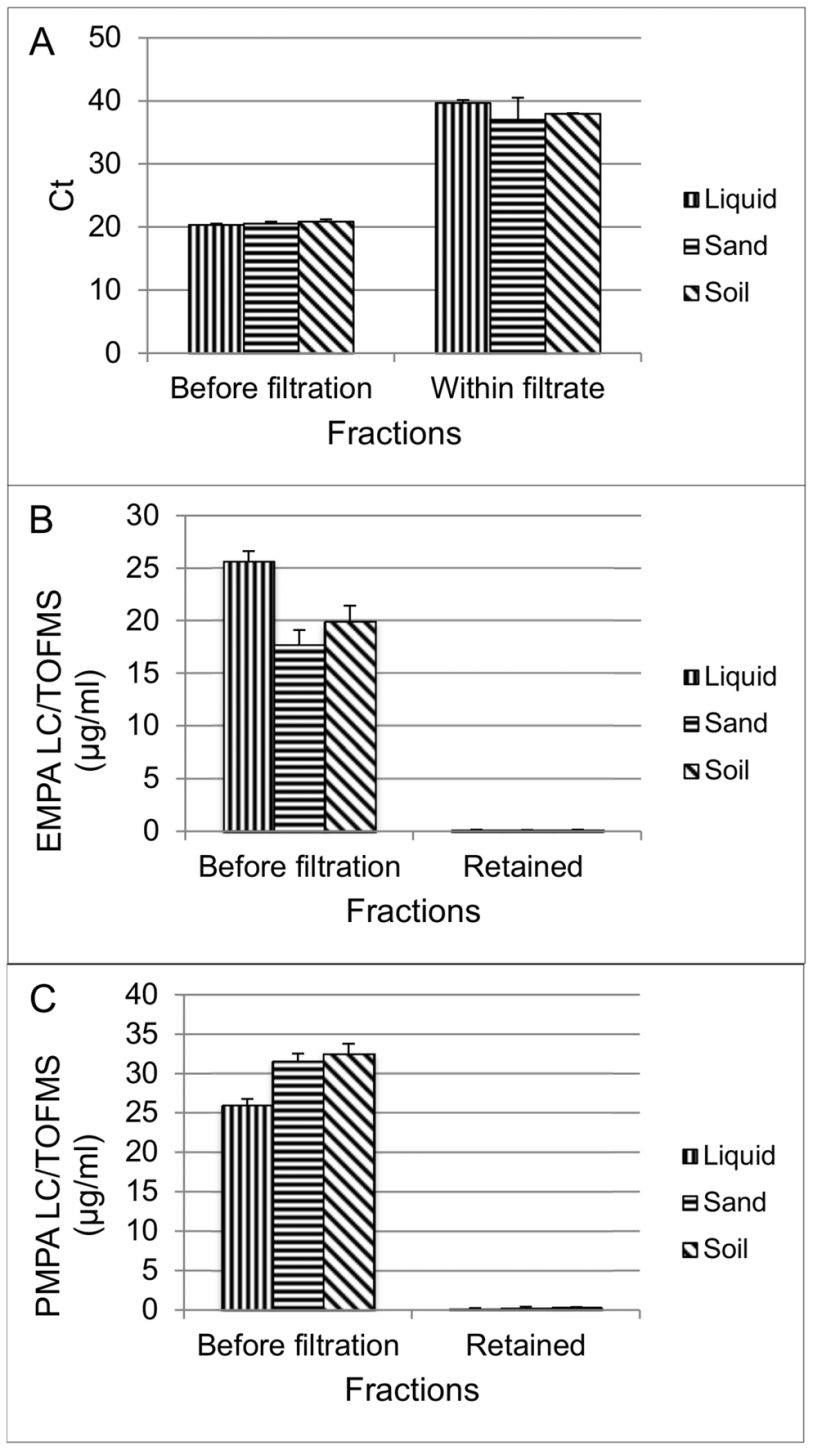
MS2 titer and EMPA or PMPA concentrations after separation of artificial CB mixed sample n°2. The CB mixed sample n°2 consisted of a fixed amount of MS2 bacteriophage (5×10^7^ genome copies in 20 µL) mixed with 25 µg/ml of EMPA and PMPA and either spiked in a liquid, sand or soil matrix. After filtration through a 30 kD filtration device, MS2 bacteriophage titers and EMPA and PMPA concentrations were measured by qPCR and LC-TOFMS, respectively. RNA extraction was carried out using NucliSens kit. (A) Mean Ct and SD (n = 3) values in the filtrate and in the unfiltered control. (B) and (C) Average concentrations and SD (n = 3) of EMPA and PMPA compounds in the filter retained fraction and in the unfiltered control.

CB mixed sample n°2 contained a fixed amount of bacteriophage MS2, EMPA and PMPA spiked either in a liquid, sand or soil matrix. After filtration through the 30 kD cut-off filters, nearly complete filter retention of the bacteriophage MS2 was achieved (Fig. 6A). The qPCR Ct values of MS2 extracted RNA from liquid, sand or soil filtrates were consistently higher than the assay intercept value with a lower limit of the 95%/95% tolerance interval of 31.82. Log_10_ reduction higher than 4 was obtained for each matrix which is corresponding to a retention capacity higher than 99.99%. Considering the chemical compounds, a high proportion of their initial concentration was recovered from the filtrate. 95%/95% tolerance intervals of (0.33–1.02), (0.10–0.91), and (0.00–1.59) were calculated indeed for PEG-400, EMPA, and PMPA, respectively from the retained fraction (Fig. 6B and 6C).

## Discussion

A rapid ultrafiltration-based procedure was designed and assessed to efficiently separate CB mixed samples while keeping unaltered the biological and chemical integrity of each component. The aim was to allow a safe analysis and identification of biological and chemical compounds through selective removal and recovery of biological agents on a filter membrane and distribution of the chemical agents in the filtrate. Whereas bacterial spores or bacteria filtration on the basis of the particle size is common [17], ensuring an efficient filtration of nanosized biological particles such as viral agents appears far more challenging [18].

This is in fact the first report using ultrafiltration as a method for separating CB mixed samples while precisely assessing the separation efficiency with quantitative performance indicators. Two previous reports described the use of filtration as a method for recovering spores of *Bacillus* from suspicious powdery and environmental matrices or for quantifying and detecting *Salmonella* in biological samples [19], [20]. However, none of these reports addressed the issue of separating CB mixed samples nor did they assess nanosized biological agents.

In this study, the robustness and efficiency of the current separation method was demonstrated with RNA (MS2) virus, chemical PEG-400 water soluble molecule and degradation products of nerve agents VX and soman (GD). Two distinct CB mixed samples were prepared by mixing MS2 either with PEG-400 or with EMPA and PMPA in a simple liquid sample matrix or in more complex matrices like sand or soil. Witnessing a high efficiency, separation of both CB mixed samples by ultrafiltration resulted in retaining more than 99.99% of viral simulants on the filter membrane whereas ∼99% of the chemical agents were recovered in the filtered fraction.

Besides filtration efficiency, the current CB mixed sample model and quantitative performance indicators confirm the many benefits and advantages of this ultrafiltration method in terms of reproducibility, simplicity, rapidity and flexibility as well as its application to nanosized particles and complex matrices. The TAT required to achieve a complete separation process of CB mixed samples within simple and complex matrices was less than 10 minutes, which is alike the TAT required to carry out a dual filtration process for recovering bacterial spores from environmental and powdery samples [19]. At last and as demonstrated here both with liquid and complex matrices, the current method is not only able to recover spores and vegetative bacteria but also nanosized biological particles like bacteriophages MS2. This is an essential finding in terms of improvement of biosafety issues directly related to the potential presence of life-threatening viral particles in samples to be handled and prepared.

Furthermore, the current separation process also efficiently isolated two nerve agent byproducts which are water soluble. Nonetheless, it is worth noting that most chemical warfare agents are water-insoluble requiring therefore organic solvents for the extraction step. Overcoming this limitation would require the use of filtration membranes that are unaffected by organic solvents. Some preliminary tests performed on various types of membranes indicate that those made of inorganic aluminum oxide are extremely resistant to dichloromethane (methylene chloride or DCM). This is interesting considering that DCM is an organic solvent frequently used to extract chemical warfare agents from solid matrices, and paves the way for further improvement of this separation method.

Comparing the results obtained respectively with each matrix, the viral recovery efficiency was strikingly poor when assessing results from the soil matrix despite using identical extraction parameters as for liquid and sand matrices. Only 21% of the bacteriophage MS2 spiked in a soil matrix could indeed be recovered. To maximize this yield, glycine was used as recovery buffer with substantial improvement of MS2 recovery up to 60% of initial amount, in line with previous methods used to recover enteric virus from soil matrices [20]. Decreased virus recovery and nucleic acid extraction efficiency, when processing soil samples, is probably best explained by a strong viral adherence to a wide variety of solid substrates in this type of matrix. Indeed, results of recovery efficiency after spiking AcNPV, an enveloped virus, in sand or soil matrices remained also disappointing even after optimization.

Considering the first goal of this study (i.e., improved separation method for safe analysis and reliable identification of CB agents), the current separation procedure is interesting. Indeed, the main challenge is to remove the entire biological components from the CB sample, hence to allow a safe analysis of chemical compounds out of biosafety level 3 (BSL3) laboratories. In the current method, more than 10^6^ of *Bacillus atrophaeus* and 10^7^ of *Bacillus subtilis* live spores were removed completely from the initial samples, irrespective of the type of filtration membrane (Table 2). A total removal of almost 10^5^ viable MS2 viral particles was also demonstrated with 30 kD filters. However, it is worth noting that few MS2 genome copies were stochastically detected by qPCR method in the filtrate, whereas Ct values, in most cases, were either undetermined or higher than the intercept value. As determined statistically using tolerance intervals, up to 10 MS2 genome copies, which corresponds to a predictive Ct value of 31.82, could be measured in the filtrate after separation with 30 kD filters. This apparent limit in filtration efficiency could either be consecutive to the presence of degraded MS2 RNA fragments in the sample passing through the filtration membrane, or could alternatively result from non-homogeneous pore size in filter membranes. In further experiments, it will therefore be interesting to assess the resulting filtration efficacy after adding a second round filtration through an additional 30 kD filter both from same and different batches. In addition, a cascade filtration process starting with a first filtration through a 0.45 µm filter, before the two consecutive filtrations on 30 kD filters, may be also considered. As suggested by current results, this successive and differential filtration process is indeed expected to retain all spores and vegetative bacteria on the 0.45 µm filter membrane and the majority, if not all, of viral particles on 30 kD filters. The most obvious advantage of this new approach would be to avoid filter clogging when the whole sample potentially containing spores, vegetative bacteria and viruses, is not pre-filtered but directly applied to 30 kD filters [21]. In the future, the current model assessing nonpathogenic nanosized biological particles like MS2 virus could be used as a quality control procedure of the filtration efficiency in triage laboratories working in real life conditions.

Another additional practical and essential aspect of the current method with respect to biosafety lies in the separation process. Separation is indeed obtained by centrifugation, using disposal centrifugal filters which can facilitate the handling of dangerous samples while being compatible with BSL3 constraints in CBRN reference laboratories. This procedure reduces indeed considerably the risks of leakage and contamination of the working space as would be the case if alternative separation systems based on disc and syringe filters are used. In that respect, a small centrifuge with removable rotor can be placed in the BLS3 glove box in such a way that the rotor can be removed and decontaminated in case of leakage, spilling or overflows of the liquid content.

Finally, the final most challenging aspect of mixed samples separation is the reliable identification of all components present in the sample. Inactivation methods used to remove partially or totally biological agents may substantially alter other potentially important components within the sample [22]–[25]. Conversely, the current filtration-based method preserves completely the whole genuine biological and chemical information contained within the original sample, hence remaining fully compatible with further detection and identification of a potential inactivation-sensitive chemical component. This is crucial to prevent the risk of false-negative results while allowing subsequent forensic analysis on biological and chemical compounds retrieved from the sample [26].

Before implementing this method in routine CBRN separation procedures, it is therefore essential to consider carefully the type of genomic material and matrix to be addressed, and to optimize the recovery parameters and conditions of the model. A representative spectrum of biological agents, particularly nanosized biological particles like viruses, should also be included in the assessment according to the application being developed. The complexity of these aspects requires an international coordination and will therefore be further addressed and developed in the ongoing joint international work project BFREE within the setting of the Joint Investment Programme on CBRN protection launched by the European Defense Agency (EDA, JIP-CBRN, BFREE project, contract A-1152-RT-GP, started in June 2013).

In conclusion, the efficacy of using ultrafiltration for fractionating a sample that is suspected to contain mixed biological and chemical compounds into its native and respective counterparts has been demonstrated by using quantitative performance indicators for both types of compounds. While allowing a separate analysis of each compound in optimal safety conditions, this method also improves the processing of unknown CBRN samples and contributes therefore to improve preparedness and consequence management in the fight against terrorism. Further work is however required for standardizing the current filtration process, validating its applicability to water-insoluble chemical warfare agents and optimizing the recovery of nanosized DNA- and RNA-biological agents from complex matrices such as soil. In that respect, the current model appears useful for further assessing a wide panel of CB mixed samples, as well as for evaluating and comparing new separation devices including those which are unaffected by organic solvents.
